# Neurotrophin receptors expression and JNK pathway activation in human astrocytomas

**DOI:** 10.1186/1471-2407-7-202

**Published:** 2007-10-31

**Authors:** Martha Assimakopoulou, Maria Kondyli, George Gatzounis, Theodore Maraziotis, John Varakis

**Affiliations:** 1 Department of Anatomy, School of Medicine, University of Patras, 26500 Patras, Greece; 2 Department of Neurosurgery, School of Medicine, University of Patras, 26500 Patras, Greece

## Abstract

**Background:**

Neurotrophins are growth factors that regulate cell growth, differentiation and apoptosis in the nervous system. Their diverse actions are mediated through two different transmembrane – receptor signaling systems: Trk receptor tyrosine kinases (TrkA, TrkB, TrkC) and p75^NTR ^neurotrophin receptor. Trk receptors promote cell survival and differentiation while p75^NTR ^induces, in most cases, the activity of JNK-p53-Bax apoptosis pathway or suppresses intracellular survival signaling cascades. Robust Trk activation blocks p75^NTR ^-induced apoptosis by suppressing the JNK-p53-Bax pathway. The aim of this exploratory study was to investigate the expression levels of neurotrophin receptors, Trks and p75^NTR^, and the activation of JNK pathway in human astrocytomas and in adjacent non-neoplastic brain tissue.

**Methods:**

Formalin-fixed paraffin-embedded serial sections from 33 supratentorial astrocytomas (5 diffuse fibrillary astrocytomas, WHO grade II; 6 anaplastic astrocytomas, WHO grade III; 22 glioblastomas multiforme, WHO grade IV) were immunostained following microwave pretreatment. Polyclonal antibodies against TrkA, TrkB, TrkC and monoclonal antibodies against p75^NTR ^and phosphorylated forms of JNK (pJNK) and c-Jun (pc-Jun) were used. The labeling index (LI), defined as the percentage of positive (labeled) cells out of the total number of tumor cells counted, was determined.

**Results:**

Moderate to strong, granular cytoplasmic immunoreactivity for TrkA, TrkB and TrkC receptors was detected in greater than or equal to 10% of tumor cells in the majority of tumors independently of grade; on the contrary, p75^NTR ^receptor expression was found in a small percentage of tumor cells (~1%) in some tumors. The endothelium of tumor capillaries showed conspicuous immunoreactivity for TrkB receptor. Trk immunoreactivity seemed to be localized in some neurons and astrocytes in non-neoplastic tissue. Phosphorylated forms of JNK (pJNK) and c-Jun (pc-Jun) were significantly co-expressed in a tumor grade-dependent manner (p < 0.05). Interestingly, a statistically significant (p < 0.05) reverse relationship between Trk receptors LIs and pc-Jun/pJNK LIs was noted in some glioblastomas multiforme.

**Conclusion:**

In the context of astrocytomas, Trk receptors (TrkA, TrkB, TrkC) expression may promote tumor growth independently of grade. Furthermore, activation of JNK pathway may contribute to progression towards malignancy. Considering the fact that regional tumor heterogeneity may be a limiting factor for immunohistochemical studies, the significance of the reverse relationship between Trk receptors and pc-Jun/pJNK LIs with respect to biological behavior of human astrocytomas requires further evaluation.

## Background

Neurotrophins, a family of highly conserved polypeptide growth factors [nerve growth factor (NGF), brain derived neurotrophic factor (BDNF), neurotrophin-3 (NT-3), and neurotrophin 4/5 (NT-4/5)] play a crucial role in function of developing and adult vertebrate nervous system [1-3]. These growth factors mediate their actions through activation of two different transmembrane – receptor signaling systems: the Trk receptor tyrosine kinases (TrkA, TrkB, TrkC) and the p75^NTR ^neurotrophin receptor, a member of tumor necrosis factor (TNF) receptor superfamily [3,4].

Trk receptors promote cell survival via several intracellular signaling cascades e.g. Ras/PI-3k/Akt pathway [1-4]. In most cases, p75^NTR ^functions as a ligand-stimulated apoptotic receptor inducing the activity of the JNK-p53-Bax pathway or suppressing Ras/PI-3k/Akt activation. Robust Trk activation blocks p75^NTR ^-induced apoptosis by suppressing the JNK-p53-Bax pathway [3,4]. Recent data reveal that precursor forms of some neurotrophins (pro-neurotrophins) have the capacity to bind with high affinity to p75^NTR ^but not to Trk receptors suggesting that pro-neurotrophins can elicit apoptosis via p75^NTR ^even in cells expressing survival-promoting Trk receptors [5-8].

The major downstream target of JNK [c-Jun N-terminal kinases (JNKs)/Stress-activated protein kinases (SAPKs) MAP kinases] is the c-Jun transcription factor which is phosphorylated at serine residues 63 and 73 [9]. The axis JNK/c-Jun is considered to play a crucial role in signal transduction in the mammalian brain [10] while constitutive activation of JNK pathway has been linked with glial tumor development and progression [11].

Gliomas of astrocytic origin are the most common primary brain tumors of adults [12]. Astrocytomas accumulate genetic lesions affecting signal transduction pathways that control cell survival and differentiation [13]. These pathways are known to be implicated in the signaling cascades of neurotrophins via their receptors Trks and p75^NTR ^[14]. The aberrant expression or mutations of the genes encoding these receptors has been associated with gliomas [15-18] as well as medulloblastomas and neuroblastomas [15,19-21]. Recently, tyrosine kinase inhibitors (TKIs) have been designed to selectively inhibit the growth of gliomas [22,23].

The aim of this study was to investigate immunohistochemicaly the expression levels of neurotrophin receptors TrkA, TrkB, TrkC, p75^NTR ^and phosphorylated forms of JNK (pJNK) and c-Jun (pc-Jun) in serial sections of human astrocytomas of different grade of malignancy.

## Methods

### Tissue samples

Astrocytic gliomas (n = 33) were obtained from patients who had undergone surgery at Department of Neurosurgery of the Patras University Hospital (Patras, Greece) from 1991 to 1996. The formalin-fixed, paraffin-embedded archival tissue blocks were retrieved, and the matching hematoxylin and eosin (H & E) – stained slides were reviewed and screened for representative tumor regions by a neuropathologist. Tumors were evaluated by routine methods for histopathology including immunohistochemical staining for glial fibrillary acidic protein (GFAP) and Ki-67 index as proliferation marker and graded according to the diagnostic criteria of the WHO classification system [12]. The tumor characteristics and clinical features of the 33 patients are illustrated in Table 1.

**Table 1:** Trk receptors, pc-Jun and pJNK expression in human astrocytomas of different grade of malignancy.

**Case**	**Sex**	**Age**	**Histology/Grading**	**Prior treatment**	**pc-Jun**	**pJNK**	**panTrk**	**TrkA**	**TrkB**	**TrkC**
1	F	32	Diffuse fibrillary astrocytoma/II	No	0	ND	70	ND	0	0
2	M	56	Diffuse fibrillary astrocytoma/II	No	2	2	50	ND	30	60
3	F	38	Diffuse fibrillary astrocytoma/II	No	0	0	0	0	0	0
4	M	24	Diffuse fibrillary astrocytoma/II	No	10	10	10	10	5	5
5	M	11	Diffuse fibrillary astrocytoma/II	No	2	2	70	50	ND	ND
6	F	15	Anaplastic astrocytoma/III	No	0	0	2	1	5	0
7	M	62	Anaplastic astrocytoma/III	No	60	40	ND	50	60	40
8	M	56	Anaplastic astrocytoma/III	No	0	0	20	10	1	ND
9	M	46	Anaplastic astrocytoma/III	No	1	0	70	60	5	5
10	M	30	Anaplastic astrocytoma/III	Yes	2	ND	5	ND	ND	ND
11	M	35	Anaplastic astrocytoma/III	No	0	0	5	2	1	0
12	F	40	Glioblastoma multiforme/IV	Yes	0	0	0	0	ND	ND
13	M	19	Glioblastoma multiforme/IV	No	10	10	30	60	30	0
14	M	65	Glioblastoma multiforme/IV	No	90	90	20	ND	30	ND
15	F	57	Glioblastoma multiforme/IV	No	50	50	10	ND	ND	ND
16	F	53	Glioblastoma multiforme/IV	Yes	70	0	10	ND	ND	0
17	M	67	Glioblastoma multiforme/IV	No	70	0	1	1	0	0
18	F	60	Glioblastoma multiforme/IV	Yes	10	10	50	ND	ND	50
19	M	56	Glioblastoma multiforme/IV	No	70	90	80	ND	80	80
20	F	44	Glioblastoma multiforme/IV	No	0	90	ND	30	1	10
21	M	67	Glioblastoma multiforme/IV	No	80	80	10	20	10	5
22	M	60	Glioblastoma multiforme/IV	No	20	80	90	ND	60	75
23	F	59	Glioblastoma multiforme/IV	No	70	70	80	ND	20	20
24	M	25	Glioblastoma multiforme/IV	Yes	5	0	40	ND	10	10
25	F	66	Glioblastoma multiforme/IV	No	90	40	60	60	30	10
26	M	61	Glioblastoma multiforme/IV	No	25	10	90	ND	20	20
27	F	67	Glioblastoma multiforme/IV	No	60	10	90	ND	60	5
28	M	42	Glioblastoma multiforme/IV	No	0	0	70	70	ND	ND
29	M	47	Glioblastoma multiforme/IV	Yes	30	0	50	50	ND	ND
30	F	65	Glioblastoma multiforme/IV	No	50	70	90	ND	70	2
31	M	53	Glioblastoma multiforme/IV	No	70	60	30	ND	ND	ND
32	F	55	Glioblastoma multiforme/IV	No	60	40	30	ND	50	30
33	M	77	Glioblastoma multiforme/IV	No	1	0	30	ND	0	0

ND: Not detected due to lack of serial sections.

### Antibodies

Anti-pan-Trk (B-3) (dilution, 1:500), a mouse monoclonal antibody that reacts with a highly conserved carboxy terminus of TrkA gp140 and is reactive with TrkA, TrkB, and TrkC receptors, anti-TrkA (763), a rabbit polyclonal antibody against a carboxy terminal epitope of human TrkA (amino acids 763–777) (dilution, 1:100), anti-TrkB (794) (dilution, 1:100), and anti-TrkC (798) (dilution, 1:100), rabbit polyclonal antibodies against carboxy terminal peptides (794–808 and 798–812 residues respectively) of gp145TrkB or gp145TrkC proteins were purchased from Santa Cruz Biotechnology (Santa Cruz, CA). Antibodies recognize the full-length isoforms of Trk receptors and the catalytic intracellular tyrosine kinase domain. According to the manufacturer, antibodies do not cross react with each other. Anti-phospho-c-Jun (KM-1) (dilution, 1:100), a mouse monoclonal antibody which detects c-Jun only when it is phosphorylated on Ser^63 ^and anti-phospho-JNK (G-7) (dilution, 1:100), a mouse monoclonal antibody directed against the carboxy terminus of JNK1 phosphorylated on Thr^183 ^and Tyr^185 ^(identical to corresponding JNK2 sequence), were also purchased from Santa Cruz Biotechnology (Santa Cruz, CA). A mouse monoclonal anti-nerve growth factor receptor (p75^NGFR^) antibody (dilution, 1:500) directed against amino acids 1–160 of extracellular domain of p75^NTR ^was purchased from Lab Vision Corporation (Fermont, CA).

### Immunohistochemistry

Immunohistochemical studies were performed on 4 μm thick serial sections. A standard biotin-streptavidin peroxidase procedure (StrAvigen MultiLink, Super Sensitive Immunodetection System B-SA; Biogenex, San Ramon, CA) for detection of pan-Trk, p75^NTR^, pc-Jun and pJNK and an anti-mouse/rabbit poly HRP IHC Detection System (Chemicon International, Temecula, CA) for detection of TrkA, TrkB and TrkC were used according to manufacturers' specifications. In brief, antigen retrieval was performed by placing the unstained slides in 0.01 mM citrate buffer (PH 6.0) and treating them in a microwave oven for 10 min. Overnight incubation at 4°C was performed for anti -pan-Trk, -p75^NTR^, -pc-Jun and -pJNK primary antibodies and 1 hour at room temperature incubation was performed for anti-TrkA, -TrkB and -TrkC primary antibodies. Antigen-antibody reaction was visualized routinely using the peroxidase enhancer as a chromogen. Mayer's hematoxylin nuclear staining was used as a counter stain. Negative controls included unrelated primary antibodies. As positive controls, specimens of inflammatory intestine known to express Trks and p75^NTR ^[24] and glioblastoma specimens known to express pc-Jun and pJNK [11] were used.

### Analysis of staining and statistical methods

The results of immunohistochemical staining were evaluated independently by two observers. Using a final magnification of 400× (objective × eyepiece), ten non-overlapping fields were chosen at random and a total of 100 tumor cells were counted manually in every field with the aid of an ocular grid. To assess the fraction of immunolabeled cells in each case and each antibody, the labeling index (LI), defined as the percentage of positive (labeled) cells out of the total number of tumor cells counted, was determined. Labeled cells in tumor blood vessels/microvascular proliferation were excluded from the cell counts.

Nonparametric methods were used for the statistical analysis of the results. Spearman correlation was used to assess significance of relationships between different LIs and between age and LIs. Spearman correlation was also used to assess significance of relationships between LIs and grade. Wilcoxon rank test was used to compare LIs between male and female. P values <0.05 were considered significant. Statistical analyses were carried out using the SPSS package (version 12.0).

## Results

### Trk expression in astrocytomas

**Figure 1:**
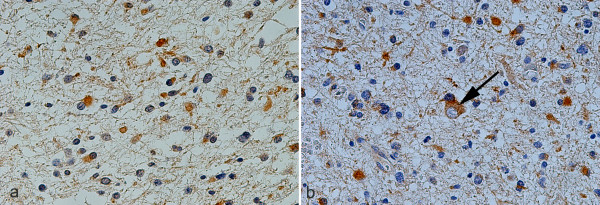
TrkB (a) and TrkC (b) immunoexpression in homologous fields of immediately adjacent sections of diffuse fibrillary astrocytoma (grade II). Note the cellular distribution of TrkC receptor in a morphologically indistinguishable cell (arrow) from the nearby cells, ×400.

**Figure 2:**
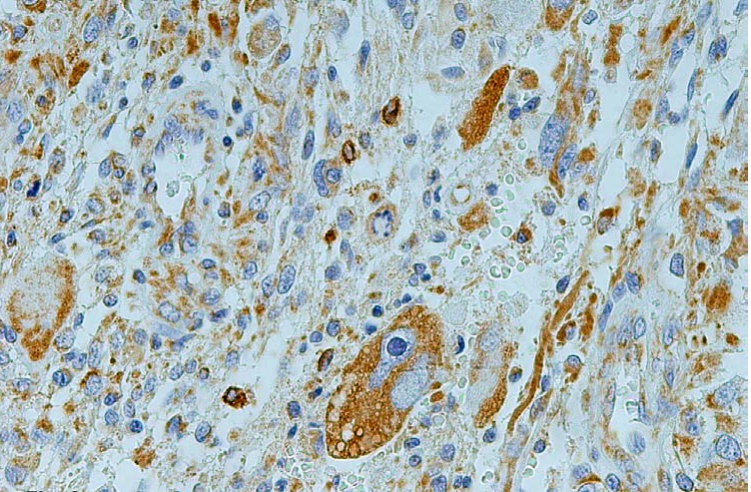
Strong TrkA granular cytoplasmic immunostaining in giant cells of glioblastoma multiforme (grade IV), ×400.

Moderate to strong Trk immunoreactivity was found in the majority of astrocytomas included in this study. Trk immunoreactivity was cytoplasmic with granular appearance (Figures 1, 2). The majority of the tumors exhibited areas with focally immunoreactive tumor cells, corresponding to 1–90% of the cell population while other areas were entirely immunonegative. The rest of the tumors displayed scattered immunoreactive tumor cells.

In diffuse fibrillary astrocytomas (WHO; grade II) reactive astrocytes showed strong immunoreactivity for all Trk receptors. Regarding grade IV tumors (glioblastomas multiforme), Trk-positively stained cells had a tendency to aggregate around tumor blood vessels or in the vicinity bordering tumor necrosis. Elongated, bipolar cells as well as giant cells (Figure 2) were intensely stained for all Trk receptors. In the majority of tumors, in immunopositive areas, the endothelium of tumor capillaries as well as florid angioproliferative changes and/or proliferations of smooth muscle cells showed conspicuous immunoreactivity for TrkB receptor. In the apparently "normal tissue" of peritumoral region found in some cases the Trk-immunoreactivity localized in some neurons and astrocytes. Blood capillaries were non reactive.

There was no statistically significant correlation between Trk expression and histological grade (Spearman correlation of grade with panTrk = 0.22, p = 0.22, Spearman correlation of grade with TrkA = 0.27, p = 0.30, Spearman correlation of grade with TrkB = 0.40, p = 0.05, Spearman correlation of grade with TrkC = 0.21, p = 0.31). Trk expression was not significantly correlated with age (Spearman correlation of age with panTrk = 0.27, p = 0.13, Spearman correlation of age with TrkA = 0.11, p = 0.66, Spearman correlation of age with TrkB = 0.25, p = 0.24, Spearman correlation of age with TrkC = 0.23, p = 0.27). Furthermore, Trk expression was not different by gender (Wilcoxon test: p = 0.40 for panTrk, p = 0.27 for TrkA, p = 0.52 for TrkB, p = 0.38 for TrkC).

### p75^NTR ^expression in astrocytomas

p75^NTR ^immunoreactivity was confined to cytoplasm of a small number (~1%) of scattered or small clusters of tumor cells in a few tumors. Normal tissue was totally p75^NTR ^-negative.

### Relationship between Trk and pc-Jun/pJNK expression

**Figure 3:**
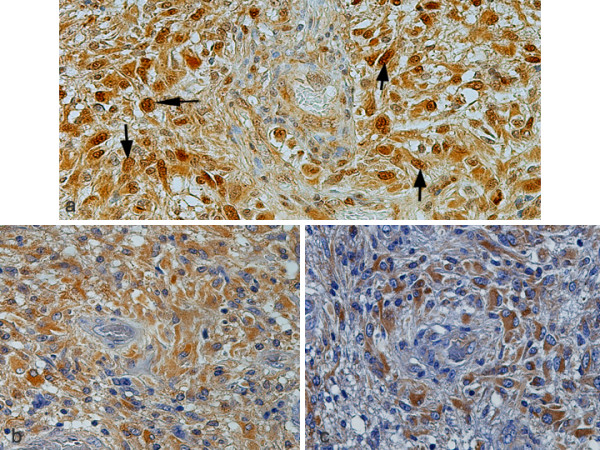
Serial sections of glioblastoma multiforme (grade IV) depicting strong nuclear (arrows) and cytoplasmic immunostaining of pc-Jun (a) and strong cytoplasmic immunostaining of neurotrophin receptors TrkB (b) and TrkC (c), ×400.

**Figure 4:**
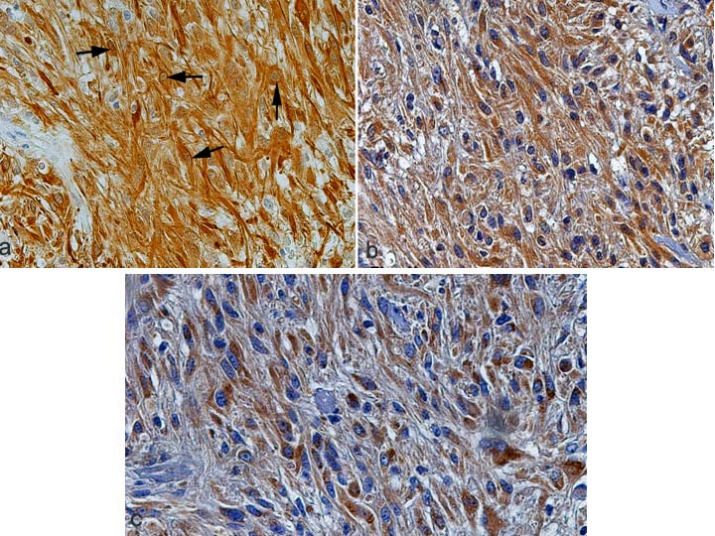
Grade IV tumor (glioblastoma multiforme) exhibiting strong nuclear (arrows) and cytoplasmic immunostaining of pJNK and strong cytoplasmic immunostaining of TrkB (b) and TrkC (c) neurotrophin receptors, ×400.

Trk receptors, pc-Jun and pJNK expression was analyzed by examining adjacent (serial) sections of each specimen. Only the nuclear immunoreactivity was evaluated for both pc-Jun and pJNK proteins. There was a statistically significant association between pc-Jun and pJNK expression (Spearman correlation = 0.57, p = 0.001). There was also a statistically significant positive correlation between pc-Jun/pJNK expression and grade of malignancy (Spearman correlation of pc-Jun with grade = 0.52, p = 0.002, Spearman correlation of pJNK with grade = 0.36, p = 0.04). pc-Jun/pJNK expression was significantly correlated with age (Spearman correlation of age with pc-Jun = 0.60, p = 0.000, Spearman correlation of age with pJNK = 0.38, p = 0.03). pc-Jun/pJNK expression was not different by gender (Wilcoxon test: p = 0.89 for pc-Jun, p = 0.83 for pJNK). Normal tissue was pc-Jun and pJNK imunonegative. Regarding co-expression of Trk receptors and pc-Jun/pJNK in the same tumor cells three patterns were noted: Trk-positive – pc-Jun/pJNK-positive cells, Trk-positive – pc-Jun/pJNK-negative cells and Trk-negative – pc-Jun/pJNK-positive cells (Figure 3, 4). Interestingly, TrkB expression was significantly correlated with pc-Jun expression (Spearman correlation = 0.60, p = 0.002) and pJNK expression (Spearman correlation = 0.63, p = 0.001). Furthermore, TrkC expression was significantly correlated with pJNK expression (Spearman correlation = 0.62, p = 0.002).

Some glioblastomas multiforme (gradeIV) showed a reverse relationship between Trk and pc-Jun/pJNK LIs. In other words, a high LI for Trk expression was associated with a low LI for pc-Jun/pJNK expression in the same tumor and vice versa (Table 1). In this subgroup of glioblastomas multiforme (grade IV) a statistically significant negative correlation between LIs for panTrk expression and pc-Jun/pJNK was found (Spearman correlation of panTrk with pc-Jun = -0.7, p = 0.01, Spearman correlation of panTrk with pJNK = -0.7, p = 0.008).

## Discussion

There is growing evidence relating neurotrophin receptors expression in pathogenesis of astrocytomas. Wang et al. (1998) have detected TrkA, TrkB and TrkC expression in neoplastic astrocytes but not in neoplastic oligodendrocytes and suggested that Trk expression may be useful for lineage marking but unrelated to any signaling cascade involved in the maintenance of a transformed phenotype [16]. On the other hand, Wadhwa et al. (2003) have found a high TrkA and TrkB expression in low grade astrocytic tumors (grade I and grade II) and a down regulation of both Trk receptors in high grade tumors (grade III and grade IV) and concluded that these receptors may contribute to progression towards malignancy [17]. Recently, Chiaretti et al. (2004) showed that there might be an association between neurotrophin receptors expression and the pathobiology of childhood low-grade astrocytomas (grade I and II) [18].

Recent data support that, in glial cells, neurotrophins, through their Trk receptors, may act as mitogens in response to injury [25] and promote cell growth in glioblastoma cell lines [26]. Thus, the overexpression of Trk receptors (independently of grade) found in astrocytomas in the present study, may indicate an accelerated downstream tumor cell survival signaling pathway and suggests a potential mechanism for malignant transformation even in low grade astrocytomas.

In contrast to gliomas, tumors of neuroepithelial origin such as medulloblastomas [19,20] and neuroblastomas [21] which express Trk receptor have a favorable prognosis. It seems that neurotrophins may activate, through their receptors, distinctly different signaling pathways in cells of different lineages.

Trk receptors are often co-expressed with p75^NTR ^and in such instances pro-apoptotic responses of p75^NTR ^e.g. activation of JNK pathway, are, in most cases, suppressed [4]. The absence of p75^NTR ^neurotrophin receptor in astrocytomas included in this study erases the possibility of apoptosis mediated by binding of pro-neurotrophins (pro-NGF and/or pro-BDNF) to p75^NTR^, even in the presence of survival-promoting Trk receptors [5-8], in these tumors.

However, JNK activation has been reported in human gliomas [11] and a grade-dependent expression of pc-Jun/pJNK was detected in the present study. It is worth noting that a reverse relationship between Trk and pc-Jun/pJNK LIs was detected in some glioblastomas multiforme (grade IV). It seems that glial cell survival may result from a balance between positive and negative regulators (modulated by activation of selective signaling pathways through tyrosine kinases and cytokines receptors). Previous data demonstrate also that cross talk between neurotrophin/Trk signal transduction axis and JNK pathway play a specific role in glial cell migration [27]. Considering the fact that gliomas are widely infiltrating tumors, future studies would elucidate the significance of the above findings with respect to biological behavior of human astrocytomas.

TrkB receptor expression detected in tumor vessels suggests that these receptors may be an important component of the angiogenic cascade required for the development of astrocytic tumors in view of recent data showing the neurotrophin-dependent proliferation of brain capillary endothelial cells [28].

Finally, tumor cells acquire the ability to activate several pathways in order to survive and neurotrophin/Trk signal transduction axis as well as JNK pathway may regulate such survival possibilities. The complexity and cross talk between different signaling cascades may limit the potential therapeutic efficacy of targeting a single molecule. Therefore, it appears necessary to develop an expanded molecular sub-classification of these tumors and to consider combinatorial treatment regimens targeted at different pathways. However, astrocytomas are strikingly heterogeneous tumors in terms of protein profile even within a single tumor which may be a limiting factor in the immunohistochemical evaluation of protein expression levels in these tumors.

## Conclusion

Trk (TrkA, TrkB, TrkC) receptor overexpression may promote tumor cell survival and enhance tumor growth while p75^NTR ^mediated apoptosis has no functional role in human astrocytomas. Activation of JNK pathway may contribute to progression towards malignancy. Further studies would elucidate the significance of the reverse relationship between Trk LIs and pc-Jun/pJNK LIs. As tumor cells activate several pathways in order to survive, future therapeutic approach may consider combinatorial treatment regimens targeted at different pathways.

## Competing interests

All authors declare that there are no financial or non-financial competing interests (political, personal, religious, ideological, academic, intellectual, commercial or any other) in relation to this manuscript.

## Authors' contributions

MA conceived of the study, carried out the design, participated in analysis of immunostaining, performed the statistical analysis, and wrote the manuscript; MK carried out the immunostaining, participated in analysis of immunostaining, and drafted the manuscript. GG supplied clinical data and reviewed the manuscript; TM supplied clinical data and reviewed the manuscript; JV carried out the pathological staging and helped in its design and critical review of the manuscript. All authors have read and approved the final manuscript.

## Pre-publication history

The pre-publication history for this paper can be accessed here:


